# Tactic Response of Shewanella oneidensis MR-1 toward Insoluble Electron Acceptors

**DOI:** 10.1128/mBio.02490-18

**Published:** 2019-01-15

**Authors:** Joseph Oram, Lars J. C. Jeuken

**Affiliations:** a School of Biomedical Sciences and the Astbury Centre for Structural Molecular Biology, University of Leeds, Leeds, United Kingdom; California Institute of Technology

**Keywords:** *Shewanella oneidensis*, extracellular electron transfer, flavin, insoluble electron acceptors, microbe-mineral interactions, motility, respiration, taxis, tracking microscopy

## Abstract

Exoelectrogenic bacteria are defined by their ability to respire on extracellular and insoluble electron acceptors and have applications in bioremediation and microbial electrochemical systems (MESs), while playing important roles in biogeochemical cycling. Shewanella oneidensis MR-1, which has become a model organism for the study of extracellular respiration, is known to display taxis toward insoluble electron acceptors, including electrodes. Multiple mechanisms have been proposed for MR-1’s tactic behavior, and, here, we report on the role of electrochemical potential by video microscopy cell tracking experiments in three-electrode electrochemical cells. MR-1 trajectories were determined using a particle tracking algorithm and validated with Shannon’s entropy method. Tactic response by MR-1 in the electrochemical cell was observed to depend on the applied potential, as indicated by the average velocity and density of motile (>4 µm/s) MR-1 close to the electrode (<50 µm). Tactic behavior was observed at oxidative potentials, with a strong switch between the potentials −0.15 to −0.25 V versus the standard hydrogen electrode (SHE), which coincides with the reduction potential of flavins. The average velocity and density of motile MR-1 close to the electrode increased when riboflavin was added (2 µM), but were completely absent in a Δ*mtrC*/Δ*omcA* mutant of MR-1. Besides flavin’s function as an electron mediator to support anaerobic respiration on insoluble electron acceptors, we propose that riboflavin is excreted by MR-1 to sense redox gradients in its environment, aiding taxis toward insoluble electron acceptors, including electrodes in MESs.

## Abstract (precis)

Previous hypotheses of tactic behavior of exoelectrogenic bacteria are based on techniques that do not accurately control the electrochemical potential, such as chemical-in-plug assays or microscopy tracking experiments in two-electrode cells. Here, we have revisited previous experiments and, for the first time, performed microscopy cell-tracking experiments in three-electrode electrochemical cells, with defined electrode potentials. Based on these experiments, taxis toward electrodes is observed to switch at about −0.2 V versus standard hydrogen electrode (SHE), coinciding with the reduction potential of flavins.

## OBSERVATION

Exoelectrogens, such as Shewanella oneidensis MR-1, are a group of microbes able to transfer respiratory electrons to extracellular and insoluble electron acceptors. They have been employed in the development of microbial electrochemical systems (MESs) and bioremediation and are implicated in biogeochemical cycling (1–6). The role of exoelectrogens in biogeochemistry, MESs, and bioremediation is underpinned by their ability for extracellular electron transfer (EET). Besides EET efficiency, performance is dependent on the exoelectrogens’ ability to migrate toward and colonize insoluble electron acceptors. There has been a steady flow of reports focused on MR-1’s tactic response to soluble electron acceptors, which can form concentration gradients (7–11), but, notably, contrasting models have been proposed for taxis of MR-1 toward insoluble electron acceptors (9, 10, 12–14).

Here, we decided to revisit the function of electrochemical potentials in the tactic behavior of MR-1 toward electrode surfaces. An experimental platform was developed that can specifically inspect taxis of MR-1 around electrodes with defined electrode potentials (in a three-electrode system), such that the redox state of extracellular redox compounds such as flavins can be controlled. The set-up was designed with the aim to analyze both individual- and population-level behaviors of MR-1 and *ΔmtrC/ΔomcA* MR-1 (*ΔmtrC/ΔomcA* MR-1 is a mutant strain deficient in outer membrane cytochromes that are required for EET).

### Tracking analysis.

Within the first 20 min after loading and sealing an electrochemical cell with MR-1, a high proportion (>70%) of bacteria displayed high motility throughout the cell with no relationship toward the electrode. This is expected as after sealing the cell, MR-1 will consume oxygen still dissolved in the medium. Between 20 and 60 min the motility gradually decreases as oxygen is consumed.

Microscope videos (at 10 FPS) were recorded of the electrochemical cell while applying various potentials to the gold working electrode. Motility of bacteria was analyzed using a tracking algorithm described in the supplemental material (Text S1). Similar to previous observations (12–14), only a subpopulation of the bacteria was observed to be motile. Here, only motile bacteria are considered, defined as bacteria with velocities >4 µm/s, and it is observed that 50% ± 10% of the bacteria are motile. Two key parameters are presented here: average velocity of all motile bacteria within an area (for bacteria with velocities >4 µm/s) and motile population density, which is the density of bacteria with mean velocities >4 µm/s in a specified area.

10.1128/mBio.02490-18.3 TEXT S1 Supplementary methods. Download Text S1, DOCX file, 1.3 MB. Copyright © 2019 Oram and Jeuken.. 2019 Oram and Jeuken This content is distributed under the terms of the Creative Commons Attribution 4.0 International license.

### Taxis is dependent on electrode potential.

**FIG 1:**
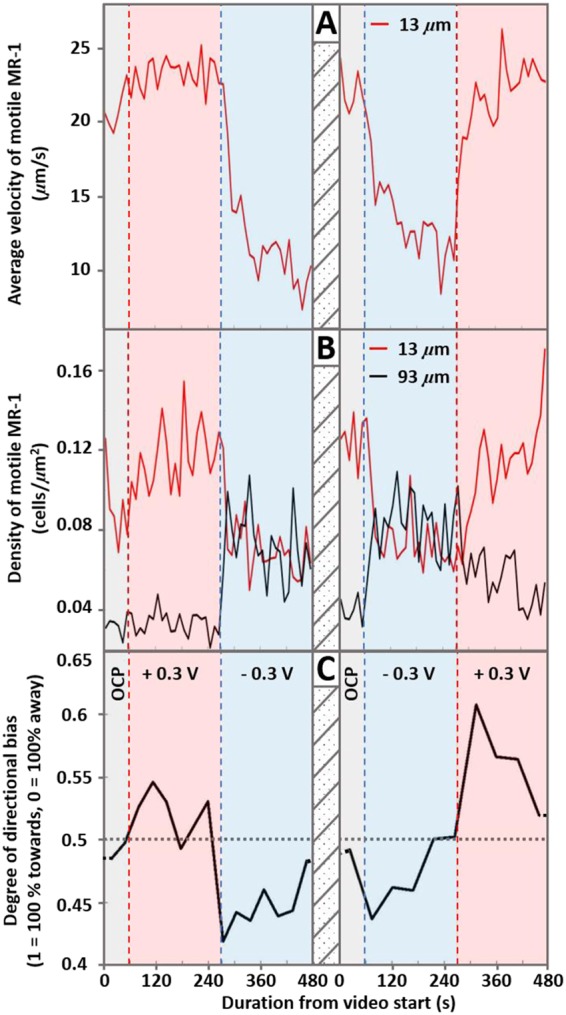
Time traces of (A) average velocity, (B) motile population density, and (C) directional bias of MR-1 in electrochemical cells during the application of different potentials. Changes in applied potential are indicated by the blue (switch to −0.3 V SHE) and red (switch to +0.3 V SHE) dashed lines and shaded areas. (A) Average velocity of motile MR-1 close to the electrode surface (between ∼3 and 23 µm, mid-distance = 13 µm). (B) MR-1 motile population density calculated for the available area close to the electrode (between ∼3 and 23 µm, mid-distance = 13 µm, red line) and the area further away from the electrode (between 83 and 103 µm, mid-distance = 93 µm, black line). (C) The average direction of all motile bacterial movement relative to the electrode in the form of a fraction, where 1 indicates a complete and direct migration toward the electrode and 0 indicates migration away from the electrode. Each data point was calculated as the weighted mean from 211 to 773 complete traces. The first video (left) was taken after ∼60 min from loading and sealing the cell with MR-1 suspension. The second video (right) was taken 30 min after the first. All samples consist of the bacteria (MR-1) grown semianaerobically in LB and diluted 5-fold with 20 mM MOPS, 30 mM Na_2_SO_4_, pH 7.4, supplemented with 50 mM lactate and 2 µM riboflavin. During recording of the first video (left), the potential remained at open cell potential (OCP, −0.18 V) for the first 60 s, after which potentials of +0.3 V and −0.3 V were applied for 210 s each. During recording of the second video (right), the potential remained at OCP (−0.18 V) for the first 60 s, after which potentials of −0.3 V and +0.3 V were applied for 210 s each.

Tracking analysis shows that when oxidative potentials are applied (0.3 V versus SHE), motile MR-1 bacteria accumulate close to the electrode (Fig. 1). These effects are stronger if an additional 2 µM riboflavin is added to the capillary cell (see Fig. S1). Importantly, *ΔmtrC/ΔomcA* MR-1 does not respond to the applied potential, indicating accumulation and motility are dependent on MR-1’s ability for EET (Fig. S1).

10.1128/mBio.02490-18.1 FIG S1 The relationship between the motile population density (top) and average velocity (bottom) as a function of the distance from electrodes poised at oxidizing (+0.3 V versus SHE, solid lines) and reductive (−0.25/−0.3 V versus SHE, dotted lines) potentials. Inset in the top right is a histogram of the bacterial velocities during the same experiment. MR-1 samples with 2 µM extra riboflavin (RF) are represented by red lines, MR-1 with no added riboflavin is represented by black lines, and the *ΔmtrC/ΔomcA* mutant strain is represented by blue lines. All samples consist of the bacteria (MR-1 or *ΔmtrC/ΔomcA*) grown semianaerobically in LB and diluted 5-fold with 20 mM MOPS, 30 mM Na_2_SO_4_, pH 7.4, supplemented with 50 mM lactate and, for the red lines only, 2 µM riboflavin. Download FIG S1, TIF file, 0.4 MB. Copyright © 2019 Oram and Jeuken.. 2019 Oram and Jeuken This content is distributed under the terms of the Creative Commons Attribution 4.0 International license.

Switching to a relatively reducing potential of −0.3 V results in a sudden drop in average velocity and motile population density close to the electrode (3 to 23 µm, red lines in Fig. 1A and B). Somewhat unexpectedly, at first glance, is the increase in motile population density further away from the electrode (83 to 103 µm, black lines in Fig. 1B). On further analysis, however, this is due to MR-1 migrating away from the electrode upon applying a reducing potential. In doing so, motile MR-1 bacteria pass through areas further away from the electrode, explaining the increase in motile population density further away from the electrode. The latter is also apparent when analyzing the directional bias of motile bacteria (Fig. 1C), which is quantified by treating the tracked trajectories as vectors with a magnitude and a directional component. Here the changes in net swimming direction can be clearly seen and coincide well with the changes in applied potential.

To validate the tracking analyses, videos were also analyzed with a method based on Shannon’s entropy, which does not require locating bacteria and their trajectories, both of which can be biased depending on the tracking algorithm used. Shannon’s entropy is the entropy for each pixel in the video based on intensity fluctuations over a specified duration. Shannon entropy values for pixels in areas of high bacterial motility will thus be higher than areas of low or no motility. Shannon’s entropy has previously been used in the analysis of time series in numerous fields, including bacterial population motility by Nisenbaum et al. (15). Qualitatively identical results were obtained using the two unrelated techniques (Fig. S2). Agreement between results obtained from tracking analysis and Shannon’s entropy confirms the validity of the analytical methods.

10.1128/mBio.02490-18.2 FIG S2 Time traces of MR-1 motile population density (top) and the mean Shannon entropy (bottom) for two regions with distances of 13 ± 10 and 93 ± 10 µm from the electrode (i.e., regions are strips of areas 3 to 23 and 83 to 103 µm from the electrode). The video was taken after about 90 min from loading the electrochemical cell with MR-1 suspension. The suspension consists of MR-1 in LB medium, diluted 5-fold with 20 mM MOPS, 30 mM Na_2_SO_4_, pH 7.4, and supplemented with 50 mMlactate and 2 µM riboflavin. In parallel to the video, the applied potential to the gold electrode was varied, starting at the OCP (−0.18 V versus SHE) for the first 60 s, followed by −0.3 V (SHE) and +0.3 V (SHE) for 210 s each. Download FIG S2, TIF file, 0.9 MB. Copyright © 2019 Oram and Jeuken.. 2019 Oram and Jeuken This content is distributed under the terms of the Creative Commons Attribution 4.0 International license.

### Tactic behavior switches at about −0.2 V.

**FIG 2:**
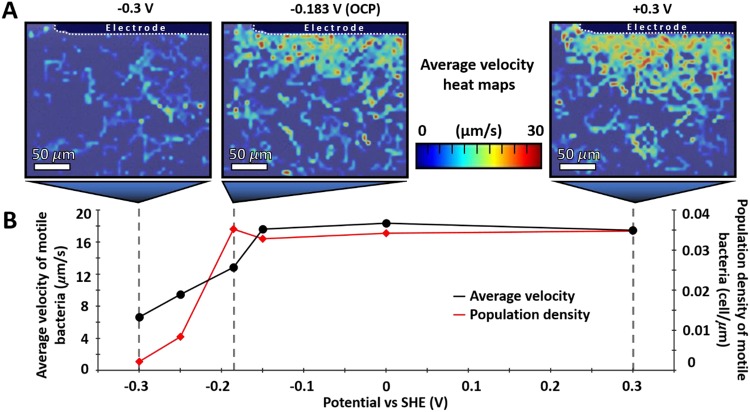
(A) Average velocity of MR-1 (presented as heatmaps) for three potentials (−0.3, −0.183, and +0.3 V versus SHE) representing the data used to calculate three of the data points shown in panel B. (B) Overlaid plots of the motile population density (red) and average velocity (black) of MR-1 within 20 µm of the electrode surface as a function of the electrochemical potential. Each applied potential was held for at least 3 min, and only data collected in the third minute were used. The data used to produce panel B were collected over two consecutive videos at the location/experiment. The first video was taken after 60 min from loading and sealing the cell with MR-1 suspension. The cell was loaded with bacteria (MR-1) grown semianaerobically in LB and diluted 5-fold with 20 mM MOPS, 30 mM Na_2_SO_4_, pH 7.4, supplemented with 50 mM lactate and 2 µM riboflavin.

By applying a series of potentials, while measuring the bacterial response through quantifiable parameters (average velocity, motile population density, and directional bias), the potential can be identified at which the change in bacterial behaviors occurs. Throughout all the video microscopy experiments carried out using MR-1, with or without added riboflavin, the shift in motile behavior is observed between −0.25 and −0.15 V. For MR-1 with added riboflavin, the window where the change occurred is narrower and between −0.25 V and −0.205 V, which coincides with the reduction potential of riboflavin (Fig. 2).

Here, tactic behavior was mostly absent in *ΔmtrC/ΔomcA* MR-1. Although in this work only riboflavin was added, it is well known that MtrC reduces other flavins besides riboflavin, in particular flavin mononucleotide (FMN) (16, 17). MR-1 is known to excrete flavins, reaching concentrations between 100 and 200 nM in “closed” electrochemical systems (17). Besides secreted flavins, flavins are found in yeast extract (18) and vitamin mixes used to prepare growth media. We estimate that in our experiments (using a one-in-five dilution of LB), even without any additional riboflavin, flavin concentrations could reach up 160 to 240 nM, explaining why tactic behavior is also observed in the absence of added riboflavin (Fig. S2).

Flavins, including riboflavin (vitamin B_2_), are a vital metabolite for MR-1, and control experiments in the complete absence of (ribo)flavin are not feasible. In spite of this, we propose that MR-1 utilizes riboflavin, and possibly other flavins, as a means to migrate toward insoluble electron acceptors (e.g., metal oxides) and initiating colonization of the surface. This function would be in addition to the multiple roles of flavins in EET respiration. First, flavins act as an electron shuttle for mediated electron transfer, enabling respiration to continue in situations where the bacterium is in a planktonic state with limited soluble electron acceptors and no insoluble electron acceptors in its immediate vicinity (17). Second, flavins act as cofactors for MtrC and OmcA, enabling enhanced direct electron transfer (16). The concentrations required for efficient energy taxis could be much lower than for substantial mediated electron transfer. We thus propose that self-secreted flavins are not only redox mediators, but also redox sensors, and could be an important factor for MR-1’s survival in electron acceptor-limited niches.

The MATLAB codes used for the tracking and Shannon entropy analysis are openly available from the University of Leeds Data Repository (https://doi.org/10.5518/409).
