# Supplementary material for: Tactic Response of Shewanella oneidensis MR-1 toward Insoluble Electron Acceptors
Source: mBio. 2019 Jan 15;10(1):e02490-18. doi: 10.1128/mBio.02490-18 (PMC6336422; doi:10.1128/mBio.02490-18)
Supplement: TEXT S1 [file mBio.02490-18-s0001.docx]

**Supplementary Methods**

**MATERIALS AND METHODS**

**Strains.** Wild type (WT) MR-1 and the double deletion *ΔmtrC/ΔomcA* (SO_1778/SO_1779) were obtained as kind gifts from J. Butt (University of East Anglia) and L. Shi (China University of Geosciences).(1)

**Growth and media.** S. oneidensis MR-1 and mutants (*ΔmtrC/omcA*) were grown on lysogeny broth (LB) plates from -80 °C glycerol stocks. Single colonies from plates no more than 3 days old were used to inoculate LB (15 mL) in a 50 mL falcon tube. With cap loosened, the cultures were incubated aerobically (30 °C, 200 rpm, 16 h) to an optical density OD_600 nm_ > 1. A small sample (~500 µL) of this culture was used to inoculate LB (50 mL) supplemented with 50 mm lactate and 25 mm fumarate in a 50 mL falcon tube. To facilitate microaerobic/semi-anaerobic conditions, the top of the tube was fastened shut and the culture was grown overnight (0 rpm, 30 °C). This set up allows for a gradual transition from aerobic to anaerobic.

After overnight semi-anaerobic incubation, cultures reached an OD_600 nm_ of approximately 0.7 and pH of 6.25–6.30. In preparation for experiments, a 2 mL sample from the 50 mL cultures was diluted 5-fold with buffer to 10 ml. The buffer used was MOPS buffer (20 mm MOPS, 30 mm Na_2_SO_4_ in H_2_O at pH 7.4) supplemented with 50 mm lactate, with or without 2 µm riboflavin.

**Electrode preparation.** High purity gold (99.99+%), silver (99.99%) and platinum (99.9+%) wires (25 µm diameter, GoodFellows, were used as working, reference and counter electrodes, respectively. Gold electrodes were modified with a self-assembled monolayer (SAMs), prepared by immersion in a 1 mm 57:43 mix of 8-mercapto-1-octanol and 8-mercato-1-octanoic acid in isopropanol for 2 days at 22 °C. Prior to use, the wires were rinsed with isopropanol and dried in air.

Ag/AgCl reference electrode wires were produced electrochemically from silver wire using a standard three electrode set-up with saturated potassium chloride as electrolyte by applying a potential of +200 mV (vs Ag/AgCl sat. KCl, Radiometer) for 60 s to the silver wire, at which point the submerged part of the wire had changed from shiny silver to a dull brown/black color.

All potentials mentioned in the results section are given versus standard hydrogen electrode (SHE). The capillary cells contain 34 mm Cl^-^ (due to a one in five dilution of LB) and hence the potential versus SHE was obtained by adding 0.308 V to the applied/measured potentials recorded using the Ag/AgCl wire reference electrode.

**Capillary electrochemical cell set up.** The capillary cell set up is depicted in **Fig. S3**. All equipment used in the set-up was autoclave sterilized where possible or treated with isopropanol for non-autoclavable items. Capillary electrochemical cells were constructed on standard 25 x 75 mm microscope glass slides (Fisher Scientific). Before use, the glass slides were treated, as per instructions, with Sigmacote^®^ (hydrophobic coating from Sigma). Glass capillary tubes (0.05 x 0.5 x 50 mm, Microslide Tube, Camlab, UK) were placed onto the center of the glass slide and kept in place with two ~3 x 15 mm pieces of Scotch tape near both ends of the capillary tube. A further, smaller capillary tube (0.02 x 0.2 x ~15 mm, Microslide Tube, Camlab, UK), shortened from its original 50 mm length, was placed inside one end of the larger capillary tube, with a ~2 mm section left out. The purpose of the smaller capillary tube is to spatially separate the counter and reference wire to prevent short circuiting. On one side of the capillary opening, with the smaller capillary protruding, a platinum wire (25 µm diameter, 40 - 50 mm long) was inserted along the gap between the outer and inner capillary, extending slightly (~1-3 mm) beyond where the end of the inside capillary was resting. Through the gap on the opposite side of the same end, a Ag/AgCl wire (25 µm diameter, 40 - 50 mm long) was inserted, parallel to the platinum wire but physically separated by the smaller capillary. Both wires extended ~15 mm into the main capillary with ~25 - 35 mm left protruding out from the capillary. If required, small adjustments can be made at this point, using tweezers and microscope, until both the Ag/AgCl and platinum wires extend by ~1 mm beyond the end of the inside capillary. Once the platinum wire (counter electrode) and Ag/AgCl wire (reference electrode) were positioned, silver conductive paint (Electrolube, HK Wentworth Ltd, UK) was used to fix the wires in place by application over the ends of the wire on the outside of the capillary in contact with the glass slide. A ~10 mm gap was left between each of the two painted areas. After ~5 min, once the silver paint had become set, conductive adhesive copper tape strips (~ 10 x 40 mm) were firmly stuck onto each painted area separately, preventing any contact between them. Most of the copper tape length protrudes out of the glass slide enabling connection to potentiostat cables via crocodile clips.


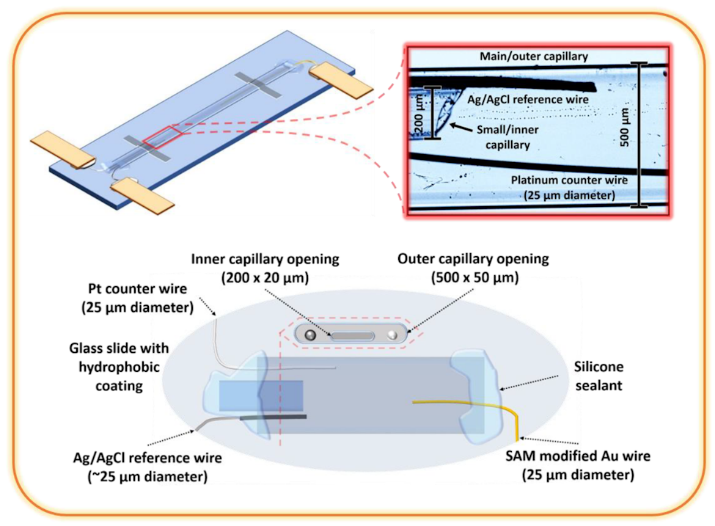
**FIG S3**: Schematic of the electrochemical capillary cell used for video microscopy cell tracking experiments.

To fill the half assembled capillary cell with bacterial suspension, approximately 1.5 mL of Bacteria suspension, prepared as described above, was transferred into a sterile 1.5 mL Eppendorf tube. The Scotch tape was removed from the end of the capillary with no wires inserted and the capillary tube prized gently away from the glass slide while still attached with Scotch tape at the other end. Under sterile conditions (i.e. under a blue flame), with the Eppendorf in an upright position and lid open, the free end of the capillary tube is submerged into the bacterial suspension just below the surface. The capillary tube was filled with the bacterial suspension through capillary force before it was lifted from the Eppendorf tube and re-fixed onto the glass slide with a thin slice of Scotch tape.

Silicon vacuum grease was applied to the opening, of both the larger and smaller capillary, at the end where Ag/AgCl reference and platinum counter wires were inserted. A SAM modified Au wire (25 µm diameter, 40 - 50 mm long) was then inserted into the unsealed opening of the main capillary, keeping ~15 - 20 mm distance between the Au wire and the reference/counter wires inserted into the opposite end. The unsealed end, with inserted Au wire, was then sealed with silicon vacuum grease and the section of Au wire extending out of the capillary, was fixed to the glass slide using silver paint, prior to sticking copper tape onto the painted area as previously described for the reference and counter wires. Crocodile clips were used to connect the copper tapes to the potentiostat (Chi604c, CH Instruments, Inc., Texas, U.S.A).

**Video tracking.** Once the capillary cell was fully prepared, the initial bacterial motility was confirmed using an inverted optical microscope (Eclipse Ti-U, Nikon Instruments, U.K.) with 40 x air objective N.A. = 0.7 and attached to an Andor Zyla 5.5 sCMOS camera (Andor Technology Ltd., U.K.). The capillary cell stability and electrical connections between wires were checked by monitoring the open circuit potential and by cyclic voltammetry (CV). CV was also used to confirm the presence of riboflavin. After initial checks, the capillary cell was left for 30 – 60 minutes to allow the bacteria to exhaust oxygen from the electrolyte. The working electrode was put in focus, (required for the video processing), preferably with the plane of focus mid-way between the floor and ceiling of the capillary interior. Videos were taken at 10 frames per second (FPS) with 2 x binning for a typical duration of 8 - 9 minutes (using the 40 x air objective and Andor Zyla 5.5 sCMOS camera), in parallel to a pre-set chronoamperometry procedure for applying a series of potential steps. Typical chronoamperometry procedures contain three steps, starting with 60 s at open cell potential, followed by two 210 s steps at alternating potentials as given in the result section. Four step chronoamperometry was also used with potentials just below and above the reduction potential of riboflavin (about -0.2 V vs SHE). After the videos were saved, electrochemical measurements (CVs at 10 and 100 mV/s) were taken. Typically, a further 2 to 3 videos along with complementary electrochemical measurements were performed on the same capillary cell. All experiments were repeated at least once and, in the case of WT MR-1, 6 times. Each repeat used a new set-up and a MR-1 colony taken from a fresh plate (<= 3 days old).

**Video processing.** To determine motility of bacteria from the bright field microscopy, videos were analyzed with a specialized tracking algorithm (see **Text S1**) based on the Crocker particle tracking algorithm(2) in MATLAB. The algorithm was designed specifically to deal with crowded, low frame rate (10 FPS) bright field videos. Manual tracking of a selection of bacteria was used to validating the tracking algorithm. After removing non-motile bacteria (defined as bacteria with velocities <4 µm/s) from the dataset (see **Text S1**), each trace was converted into vectors with start and end points and the overall direction and magnitude (distance travelled) relative to the electrode was calculated for each trajectory. Parameters such as total distance travelled and average velocities were extracted from the vectors. Datasets representing trajectories or vectors were binned according to their distance from the nearest point on the electrode surface. *Average velocity* of all motile bacteria within an area was calculated as a mean of all the individual bacterial velocities. *Motile population density* is given by the number of bacteria with mean velocities >4 µm/s in a specified area divided by that area. Together, the *average velocity* and *motile population density* of a particular area (with a given distance from the electrode) gives a measure of the motile activity of bacteria as a function of the distance from the electrode.

A secondary video processing technique was also used which has been previously validated for monitoring bacterial motility(3) and is based on Shannon’s entropy (see Scheme S4). The Shannon entropy of each pixel was calculated using 10 s (~100 frame) intervals.

**Tracker algorithm.** Cell tracking from bright-field microscope images was performed in MATLAB using a modified version of the particle tracking algorithm originally developed by Crocker(2). The MATLAB codes used for the Tracking and Shannon Entropy analysis are openly available from the University of Leeds Data Repository (http://doi.org/10.5518/409). The Crocker algorithm is tolerant to noise and capable of distinguishing between particles by size. This is useful when working with biological samples where debris may be of concern or particles/bacteria in the plane of interest are obscured by out of focus particles/bacteria. The Crocker algorithm works well with bright field images undeterred by sub optimal contrast. Particles moving in and out of focus can be an issue for tracking. For example, the algorithm may identify each reappearance of the same particle in the plane of focus as a new particle creating multiple ‘broken-up’ tracks. Crocker’s algorithm saves the location of the last known position of particles from traces which disappear and can identify the reappearing particle as the same particle if spatial and temporal translations are within a specified limit. Indeed, the Crocker algorithm works to reduce the creation of new tracks by applying a penalty to combinations in which new tracks are formed. This is an essential feature when working with highly motile bacteria such as MR-1 which are free to move in all 3 dimensions.

The actual MATLAB code used two functions (‘pkfnd.m’ and ‘track.m’) downloaded from a repository maintained by Daniel Blair and Eric Dufresne and one custom function ‘ImgFilterF.m’. ‘ImgFilterF.m’ is used to create image filters for enhancing bacterial positions, ‘pkfnd.m’ is a peak finding function for locating the origins of the enhanced features (the bacteria), retained by the band pass and 'track.m’ is a function for finding the most probable trajectories from the locations identified in a stack of images.

The custom filter ‘imgFilterF.m’ was built with MATLAB ’s fast Fourier transform implementation and was used to create a band pass filter for increasing the contrast of relevant image features whilst suppressing noise. For this, a low-pass filtered image, using a relatively low frequency cut off, is subtracted from a second low-pass filtered image, using a higher frequency cut off, from the same data. Importantly, the filter ‘ImgFilterF.m’ deals with boundaries by reflecting a section of the borders prior to transforming into Fourier space. ‘ImgFilterF.m’ does not result in any cropping or distortion of image borders and gives the user control over the band pass range.

Sections from videos (filtered image stacks), along with a table containing frame times, was processed using ‘pkfnd.m’ to obtain bacteria locations and 'track.m’ to find probable traces using the Crocker algorithm. ’track.m’ was further modified as described below. Traces were plotted and examined alongside the original video to check how well the resultant traces correlate with the actual video of swimming bacteria.


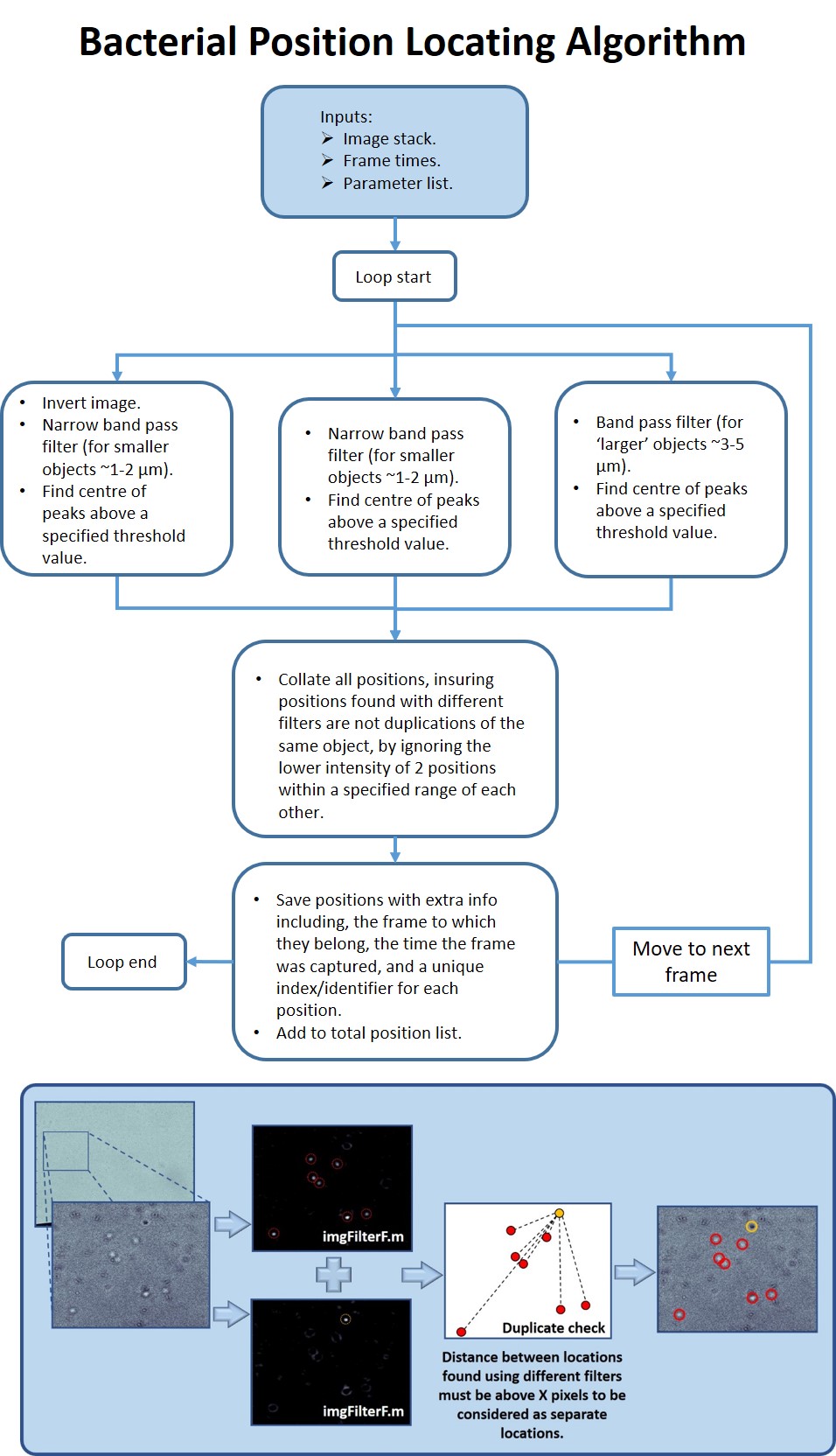


**Scheme S1** A flow diagram gives a summary of the general procedures used by the code for locating bacterial positions from a stack of images and translating the images into a list of co-ordinates with time signatures that can be input into the tracking algorithms in **Scheme S2 + S3**.


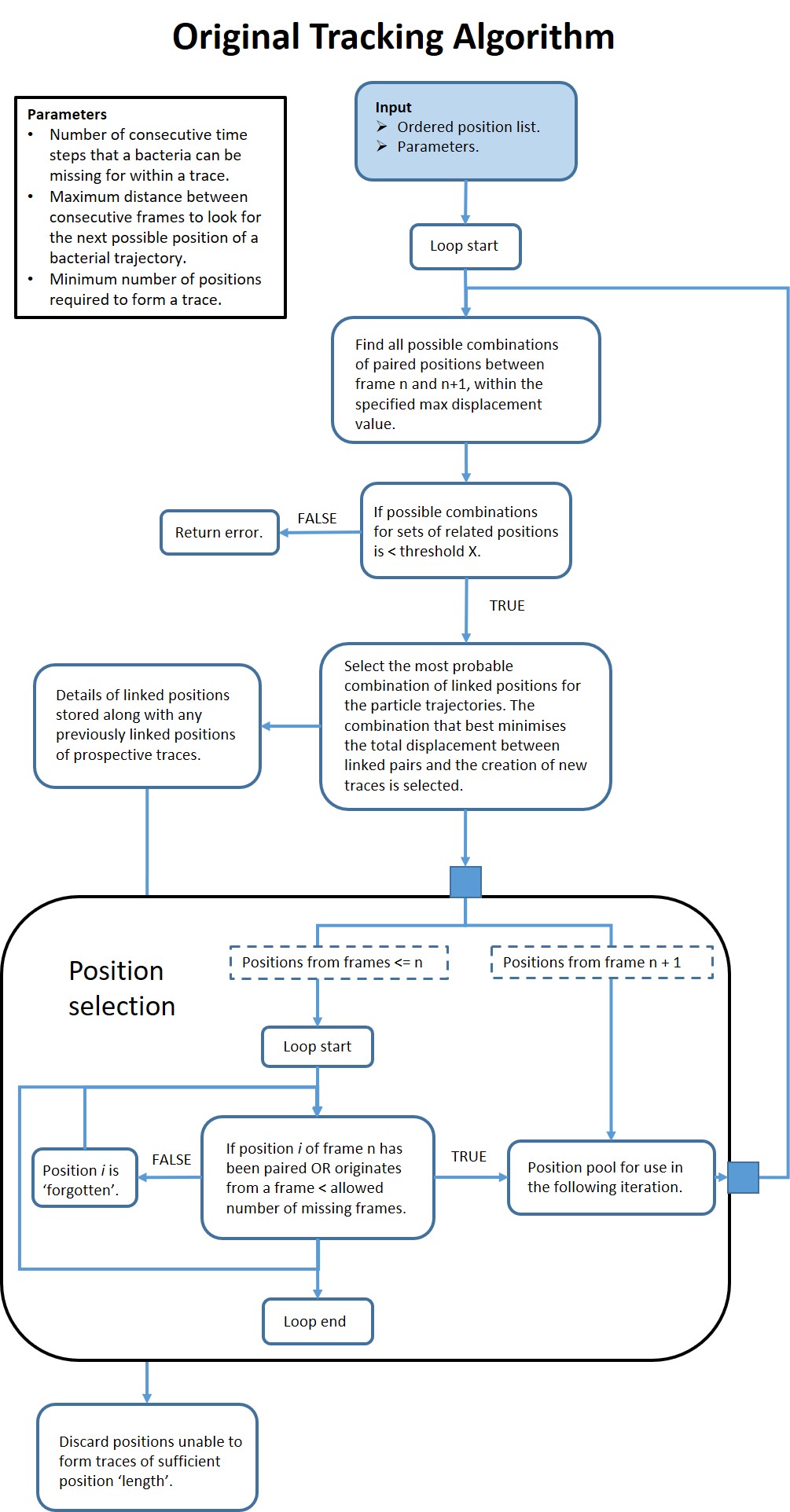


**Scheme S2:** The flow diagram gives an abstract summary of the general methodology used by the Crocker algorithm for generating the most likely trajectories from a particle/bacterial position list produced by the small algorithm outlined in **Scheme S1**.

In the case of the capillary cell experiments, bacteria are free to move within a relatively large depth range (50 μm). Depending on the plane a bacterium is in, relative to the plane in focus, it can appear as a darker or lighter feature in the image after filtering. To increase the depth in which bacteria can be located, the difference between the low and high bandpass filters can be increased (in ‘ImgFilterF.m’) and/or threshold intensity decreased (in ‘pkfnd.m’). However, this decrease in selectivity results in an increase in false positives. To overcome this, images were processed with multiple ‘narrow’ band pass filters, forming multiple processed images. This method reduced false positives while increasing the proportion of correctly located bacterial positions. Occasionally the different band pass filters identify the same bacteria, usually with a difference of a few pixels between them. To prevent duplication of bacteria positions, locations of bacteria identified within ~15 pixels between images processed with different band pass filters were processed as a single bacterium location. These changes to the process of locating bacteria were implemented as additional logic in a locator function, which contains ‘imgFilterF.m’ and ‘pkfnd.m’ as sub functions. An abstraction of the process for locating bacterial positions is described in Scheme S1 with the process for cell tracking by the Crocker algorithm in Scheme S2.

**Removing non-motile bacteria from dataset.** In all microscopy videos taken, only a sub-population of MR-1 were motile. Previous investigations involving video microscopy of MR-1 have also reported only a sub population of motile cells.(4-6) However, to ensure statistical significance of the results, the bacterial density of motile bacteria has to be sufficiently high. The penalty for processing frames with a greater bacterial density (motile and non-motile) is an increase in probability for neighbouring bacteria crossing paths. Inter-bacterial trace tangling becomes an issue once the average distance becomes equivalent to, or lower than, the distance searched for consecutive bacterial locations between frames.

This problem is aggravated when stationary objects (including non-motile bacteria) have pixel intensities close to the threshold intensity as these objects appears as transient objects (only visible in some images of the stack), leading to artefacts in the cell tracking algorithm with inadvertent effects on the resulting average velocity and average swimming direction extracted from the traces.

To form traces from fast moving bacteria, a new tracking algorithm was developed that removed non-motile objects. The new tracking algorithm incorporates a slightly modified version of the original ‘track.m’ (the Crocker algorithm) as a sub function. An abstract flow diagram of which has been laid out in **Scheme 4.3**. The custom algorithm takes substantially longer to process position lists, compared to the original algorithm, but the resulting traces are formed with considerably less artefacts. Importantly, the custom algorithm can track bacteria traveling at velocities over 250% of the max velocity trackable by the original algorithm from the same data set. It should be made clear that if a higher frame rate was used for the videos or the spatial density of bacteria was lower, the original Crocker algorithm would have worked adequately. The custom algorithms main advantage is to enable the tracking of higher velocities from videos of a sub-optimal frame rate (e.g. 10 FPS).


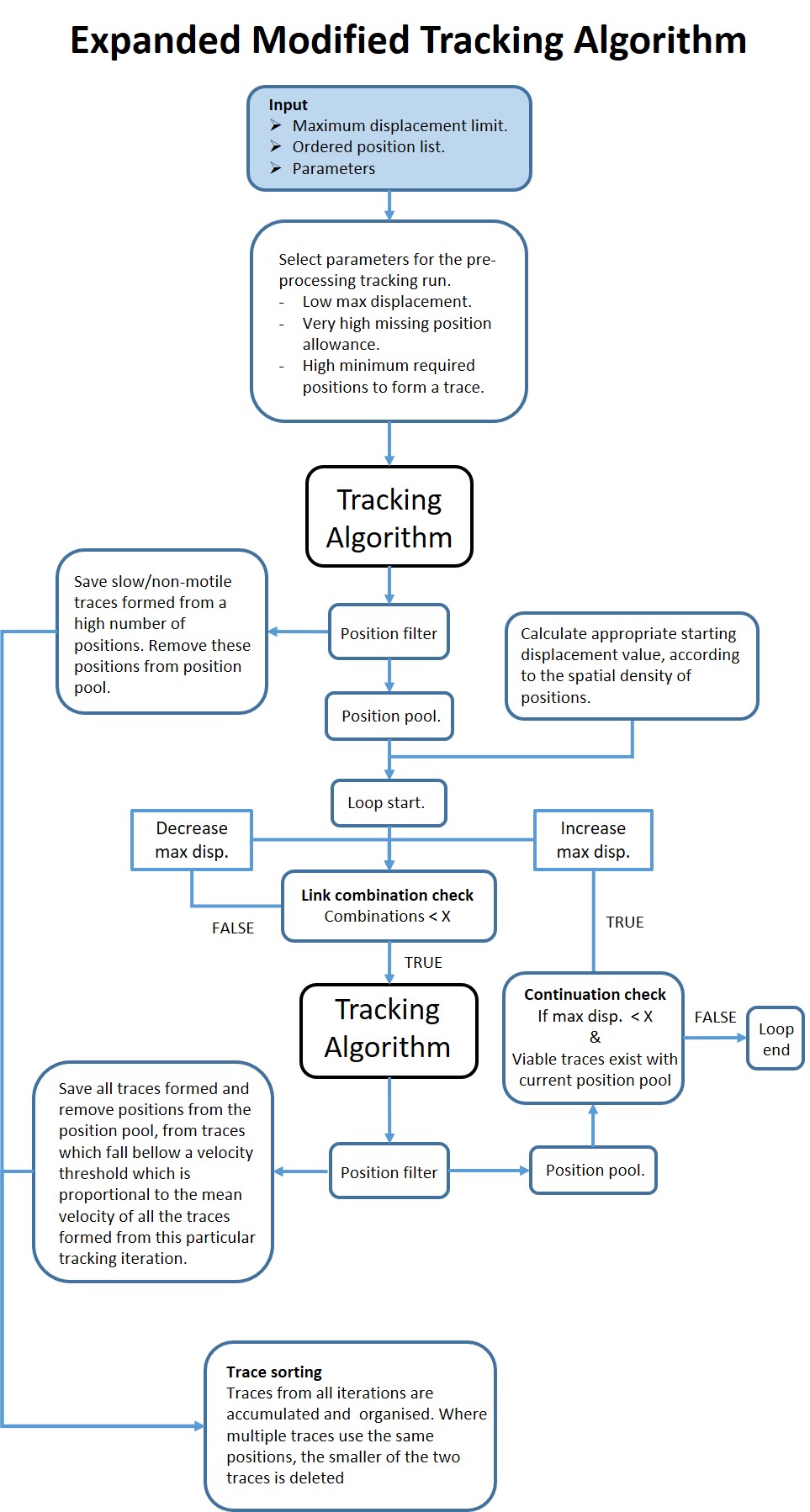


**Scheme S3:** Flow diagram overview of the custom tracking algorithm developed to address limitations with the original Matlab interpretation of the Crocker algorithm (**Scheme S2**). The flow diagram gives a summary of the procedures built around a modified version of the Crocker algorithm for generating trajectories with less artefacts and including traces from previously ‘hidden’ bacterial trajectories. Here ‘**Tracking Algorithm**’ refers to the slightly modified version of the original Crocker algorithm, which is used here as a sub function.

**Shannon’s entropy**


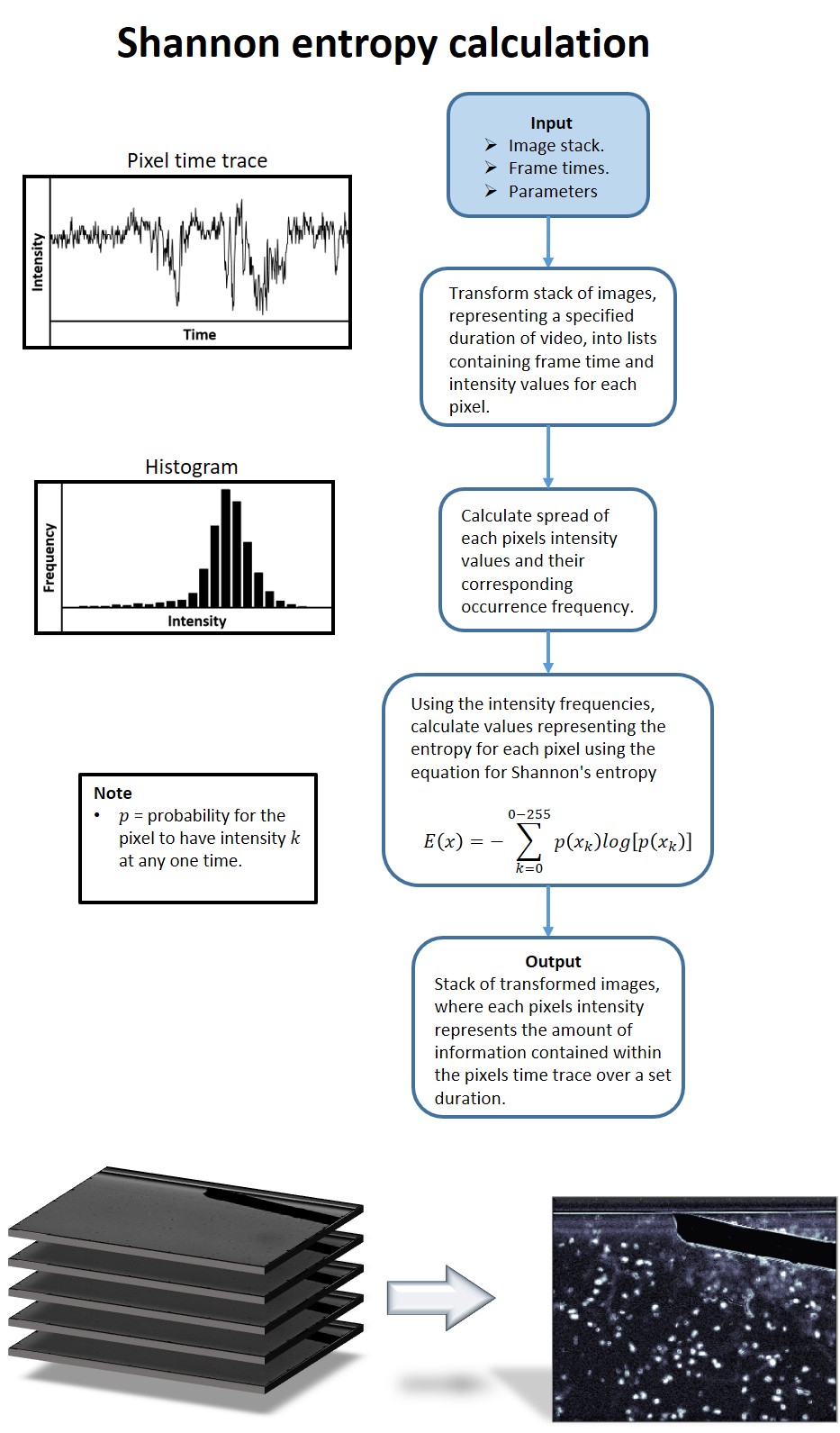


**Scheme S4.** Flow diagram overview of the custom Shannon Entropy algorithm. The flow diagram gives a summary of the general procedure used by the algorithm for generating Shannon entropy images from an image stack.

**References**

1. Marshall MJ, Beliaev AS, Dohnalkova AC, Kennedy DW, Shi L, Wang Z, Boyanov MI, Lai B, Kemner KM, McLean JS, Reed SB, Culley DE, Bailey VL, Simonson CJ, Saffarini DA, Romine MF, Zachara JM, Fredrickson JK**.** 2006. c-Type cytochrome-dependent formation of U(IV) nanoparticles by *Shewanella oneidensis*. Plos Biology 4**:**1324-1333. <https://doi.org/10.1371/journal.pbio.0040268>.

2. Crocker JC, Grier DG**.** 1996. Methods of digital video microscopy for colloidal studies. J Colloid Interface Sci 179**:**298-310. <https://doi.org/10.1006/jcis.1996.0217>.

3. Nisenbaum M, Maldonado E, Martinez Arca J, Gonzalez JF, Passoni LI, Murialdo SE**.** 2016. Video processing analysis for the determination and evaluation of the chemotactic response in bacterial populations. J Microbiol Methods 127**:**146-153. <https://doi.org/10.1016/j.mimet.2016.06.006>.

4. Harris HW, El-Naggar MY, Bretschger O, Ward MJ, Romine MF, Obraztsova AY, Nealson KH**.** 2010. Electrokinesis is a microbial behavior that requires extracellular electron transport. Proc Natl Acad Sci U S A 107**:**326-331. <https://doi.org/10.1073/pnas.0907468107>.

5. Harris HW, El-Naggar MY, Nealson KH**.** 2012. *Shewanella oneidensis* MR-1 chemotaxis proteins and electron-transport chain components essential for congregation near insoluble electron acceptors. Biochem Soc Trans 40**:**1167-U1129. <https://doi.org/10.1042/bst20120232>.

6. Kim BJ, Chu I, Jusuf S, Kuo T, TerAvest MA, Angenent LT, Wu M**.** 2016. Oxygen tension and riboflavin gradients cooperatively regulate the migration of *Shewanella oneidensis* MR-1 revealed by a hydrogel-based microfluidic device. Front Microbiol 7**:**Art No 1438. <https://doi.org/10.3389/fmicb.2016.01438>.
