# Supplementary figures and images for: Tactic Response of Shewanella oneidensis MR-1 toward Insoluble Electron Acceptors
Source: mBio. 2019 Jan 15;10(1):e02490-18. doi: 10.1128/mBio.02490-18 (PMC6336422; doi:10.1128/mBio.02490-18)

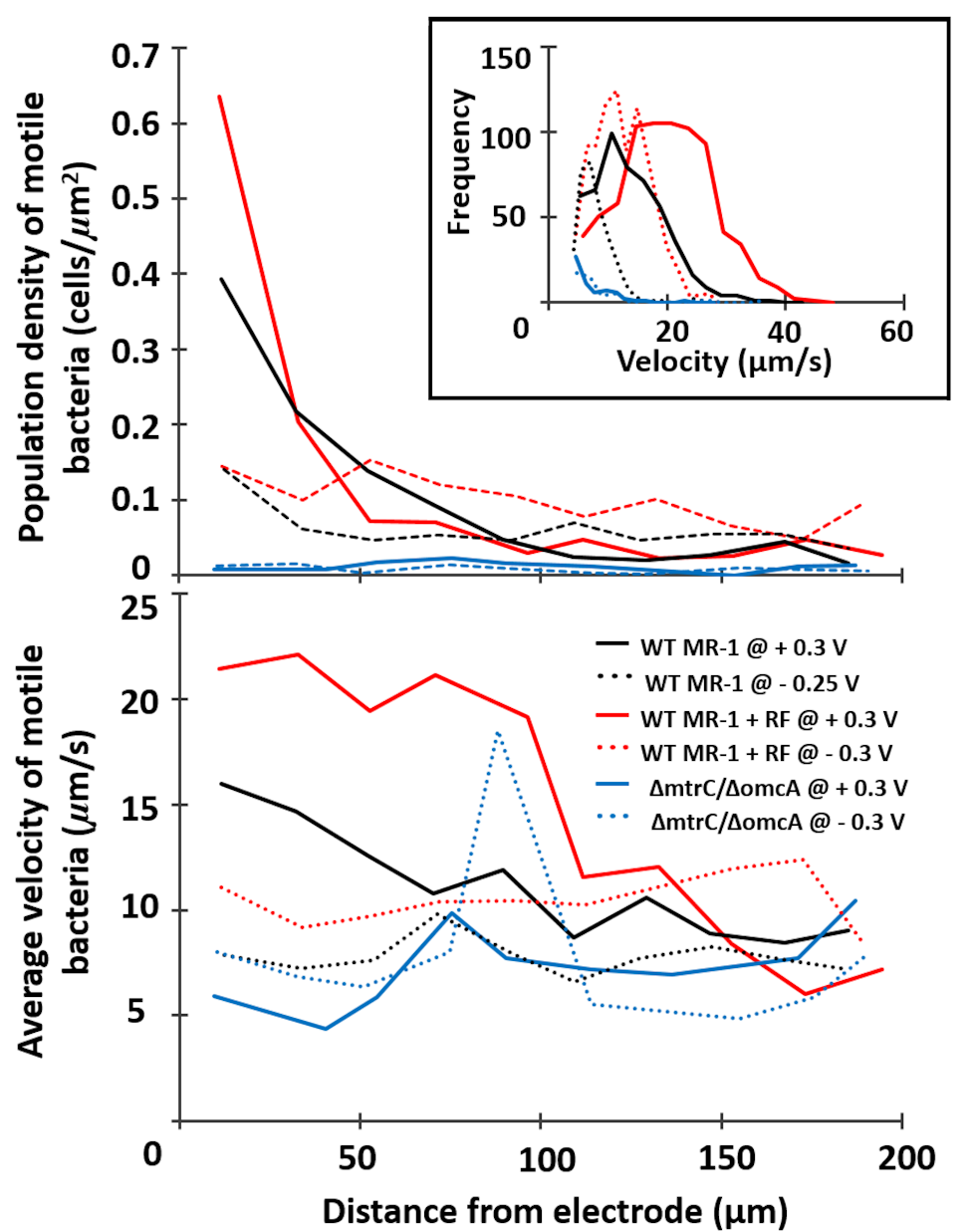

Supplement: FIG S1 [file mBio.02490-18-sf001.tif]

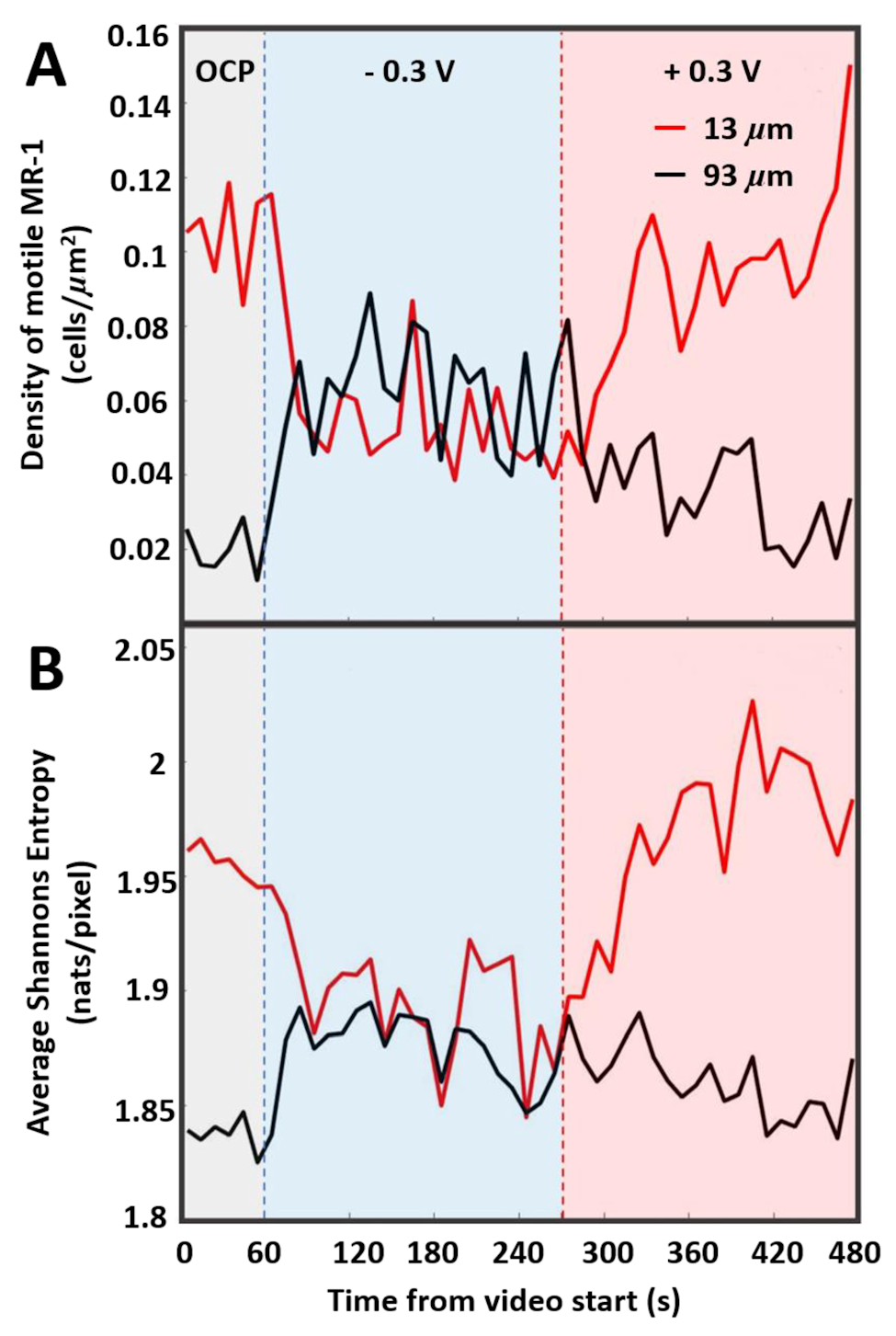

Supplement: FIG S2 [file mBio.02490-18-sf002.tif]
